# A Case Report Describing Hemophilus parainfluenza Recovered From Pleural Effusion

**DOI:** 10.7759/cureus.53004

**Published:** 2024-01-26

**Authors:** Kinnera S Urlapu, Shekhar S Bhatta, Maryam Soliman, Trupti Vakde

**Affiliations:** 1 Pulmonary and Critical Care Medicine, BronxCare Health System, Bronx, USA; 2 Internal Medicine, BronxCare Health System, Bronx, USA

**Keywords:** atypical presentation, report, case, pleural effusion, hemophilus parainfluenza

## Abstract

Hemophilus parainfluenza, a less common pathogen typically found in the oropharyngeal flora, has been associated with various clinical conditions. However, its role in pleural effusions remains scarcely documented. We present a unique case of a 42-year-old female with a history of asthma, hypertension, and obesity who presented with epigastric pain and a moderate right-sided pleural effusion. Hemophilus parainfluenza was isolated from the pleural fluid despite an atypical, asymptomatic presentation without pneumonia. Antibiotic treatment led to a positive response, highlighting the importance of recognizing Hemophilus parainfluenza as a potential causative agent in pleural effusion cases.

## Introduction

Hemophilus parainfluenza is a pathogen usually present in the normal flora of the oropharynx. Hemophilus parainfluenza has been documented as a causative agent in a spectrum of clinical conditions, encompassing pharyngitis, epiglottitis, otitis media, conjunctivitis, dental abscess, pneumonia, septic arthritis, osteomyelitis, paraspinal and epidural abscesses, peritonitis, hepatobiliary infections, meningitis, brain abscesses, as well as infections involving the urinary tract and genital region [1]. While Hemophilus parainfluenza is comparatively less prevalent than Hemophilus influenza, the diseases they incite and the resultant symptomatology exhibit notable similarities [2]. Hemophilus parainfluenza-associated pleural effusions are infrequent occurrences, and the existing body of literature on this subject is limited. Here, we present an exceptional case of pleural effusion attributed to Hemophilus parainfluenza infection in an otherwise medically uncomplicated individual.

## Case presentation

A 42-year-old female patient from Nicaragua, with a medical history of asthma, hypertension (HTN) on losartan, and obesity, presented herself to the Emergency Department (ED) complaining of epigastric pain that had persisted for three days. The patient revealed that she had been experiencing intermittent epigastric pain and bloating, notably triggered by the consumption of fatty foods, over several months. Remarkably, her symptoms emerged after she underwent a cholecystectomy procedure. Notably, she denied experiencing fever, cough, shortness of breath, chest pain, palpitations, nausea, vomiting, diarrhea, and urinary symptoms. Her vital signs remained stable upon admission to the ED. She was noted to have decreased breathing sounds in the right lung field; her physical examination was otherwise normal.

**Figure 1:**
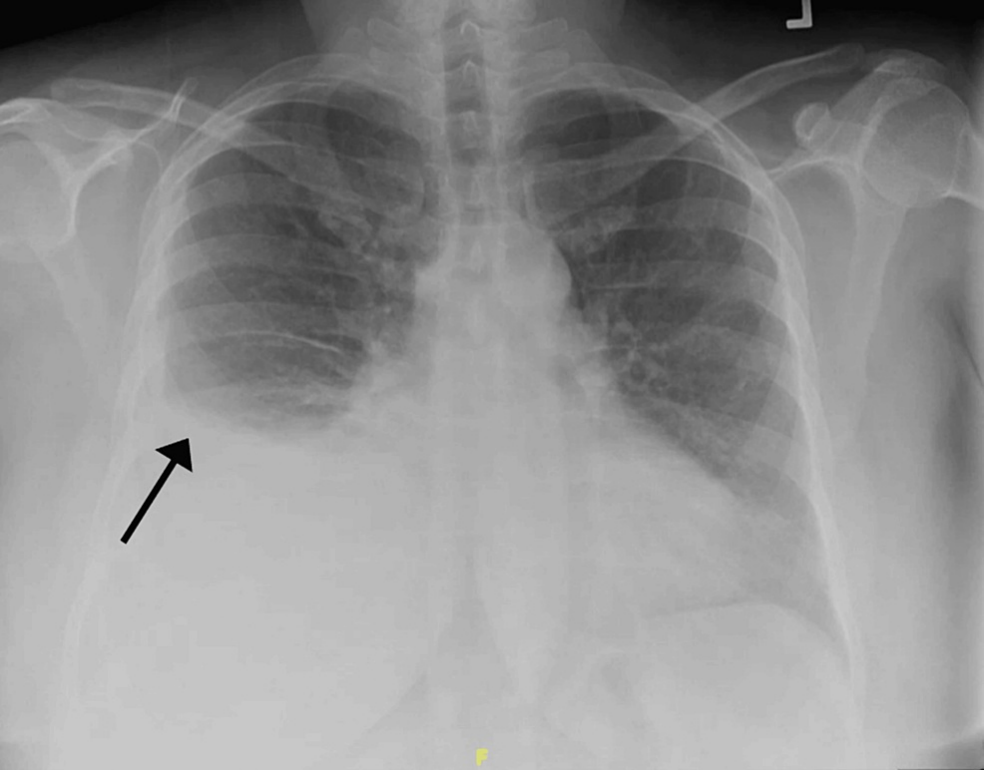
Initial Chest X-ray showing right-sided pleural effusion

**Figure 2:**
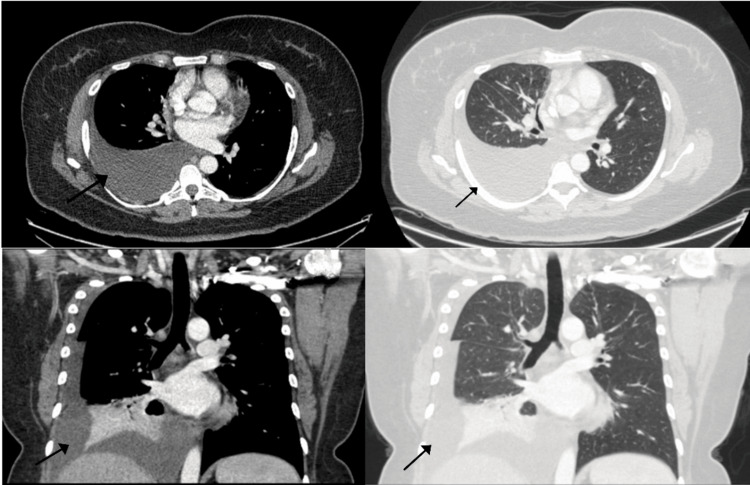
Images in the axial view (2A and 2B) showing right basal pleural effusion, note a lack of consolidation or infiltrates on the lung windows. Images in the coronal view(2C and 2D) showing right basal pleural effusion with basal atelectasis

Initial laboratory investigations highlighted a white blood cell count of 11,400/mm³, predominantly neutrophilic, alongside a lymphocyte count of 1900/mm³. Hemoglobin was measured at 14 g/dL, and the platelet count at 133,000/mm³. Of significance was an elevated c-reactive protein (CRP) level at 29.83 mg/L. Additionally, her serum creatinine level stood at 0.6 mg/dL, while albumin registered at 3.8 g/dL. A chest X-ray and a contrast-enhanced computerized tomography (CECT) scan of the chest unveiled the presence of a moderate right-sided pleural effusion accompanied by atelectasis. A chest CT revealed no mediastinal adenopathy (Figure 1, 2).

**Figure 3:**
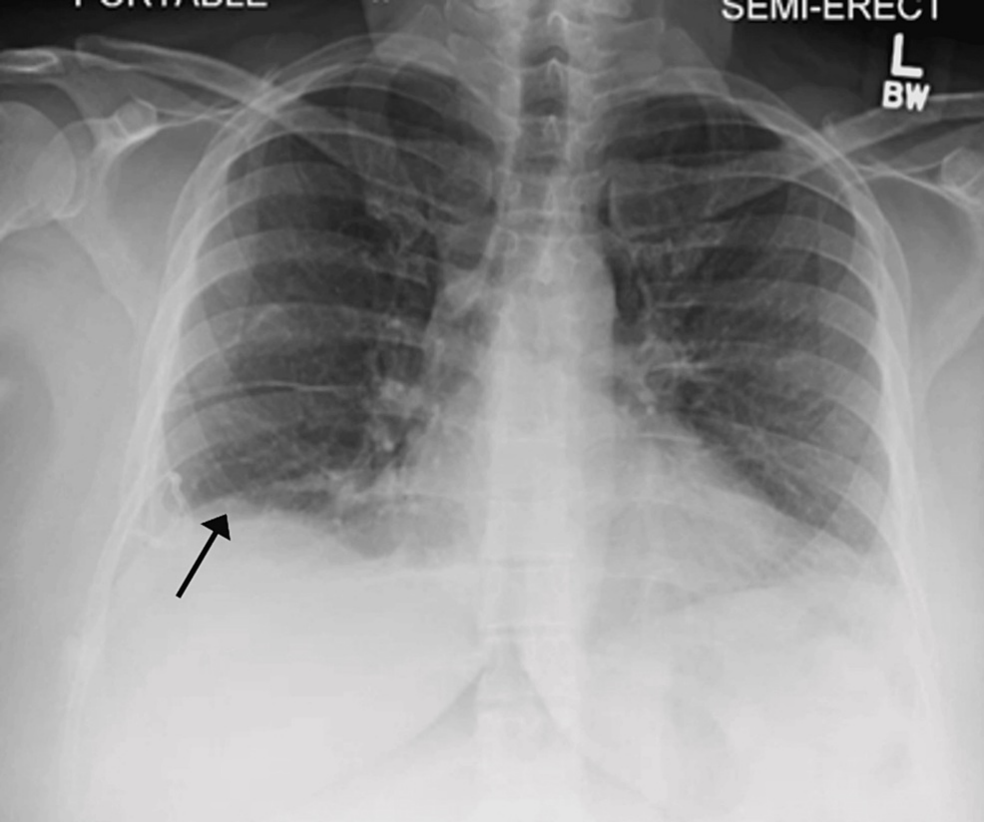
Chest X-ray post thoracentesis, showing resolving effusion. A pigtail catheter was seen in the right lower lung field

In order to further comprehend the condition, an ultrasound-guided thoracentesis was performed, successfully extracting one liter of serous fluid, following which the patient was symptomatically better. A repeat chest X-ray showed resolving effusion (Figure 3). The pleural fluid analysis unveiled a lymphocytic exudative pattern and an elevated adenosine deaminase level of 36 U/L. Notably, the mycobacterial culture of the pleural fluid and cytology yielded negative results.

**Figure 4:**
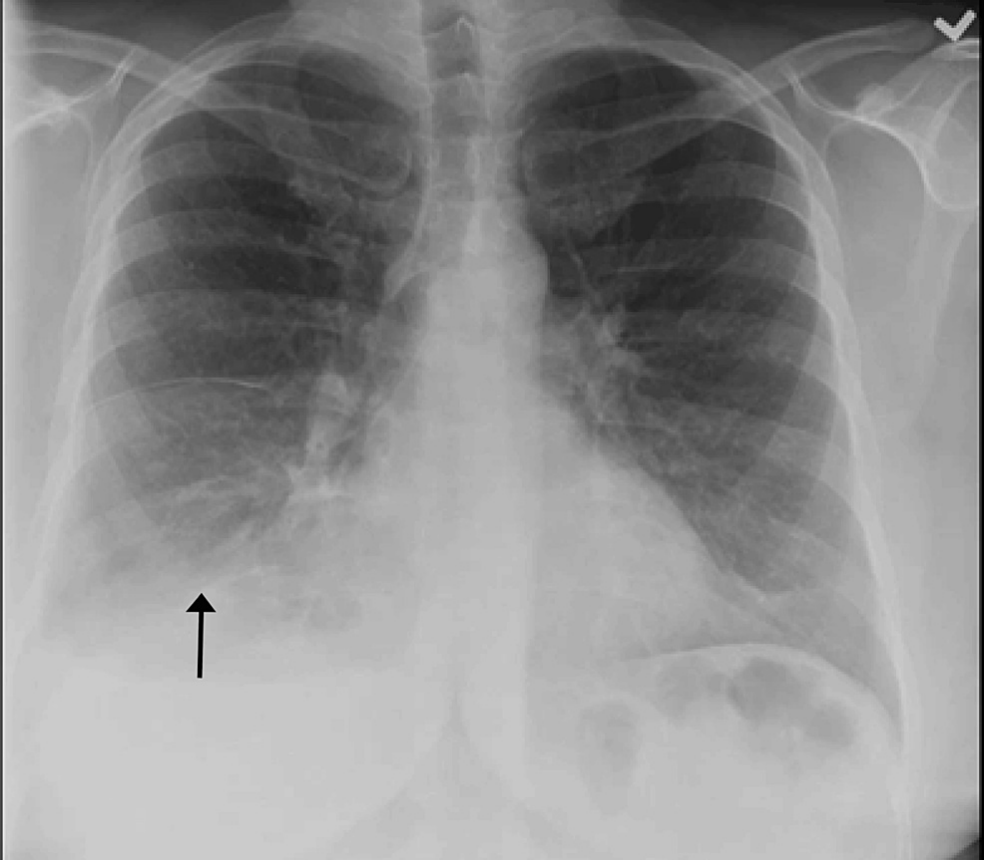
Chest X-ray showing recurrence of pleural effusion ten days later

Following her initial management, which involved administering Proton Pump Inhibitors (PPIs) leading to transient symptom relief, the patient was discharged with a plan for pulmonary follow-up without any antibiotics. However, a mere ten days post-discharge, a chest x-ray exposed a recurrence of pleural fluid accumulation (Figure 4), prompting further investigation. Notably, the culture results from the prior pleural fluid analysis revealed the presence of Hemophilus parainfluenza. As part of the diagnostic process, blood culture, HIV test, and autoimmune workup returned negative results. Intriguingly, the patient had a positive QuantiFERON test, whereas sputum and pleural fluid mycobacterial culture was negative. A subsequent transthoracic echocardiogram effectively ruled out the possibility of endocarditis.

**Figure 5:**
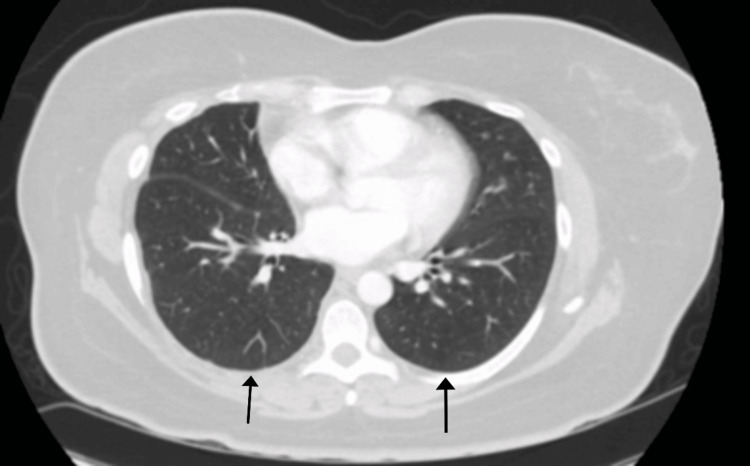
CT chest showing complete resolution of pleural effusion after 5 days of ceftriaxone

The patient was commenced on ceftriaxone 2g daily and received for five days, leading to the gradual resolution of pleural effusion. (Figure 5) She did not require repeat thoracentesis in her readmission. Upon her discharge, she was prescribed oral amoxicillin/clavulanic acid for an additional ten days for continued management. A subsequent follow-up evaluation after a week showcased a reduction in the size of the pleural effusion, and a repeat CT chest indicated a further decrease in the size of the effusion, and she remained symptom-free.

## Discussion

Pleural effusions are linked to bacterial, viral, mycobacterial, and fungal infections and non-infectious etiologies such as malignancies and systemic inflammatory disorders. Isolating Hemophilus parainfluenza from pleural fluid cultures is infrequent. To our knowledge, only two cases of Hemophilus parainfluenza-associated empyema have been reported [3,4]. In both of the previously reported cases, the patients developed empyema after the onset of pneumonia [3,4]. In contrast, our current patient did not exhibit any symptoms or indications of pneumonia and empyema; instead, they presented solely with an isolated pleural effusion.

Mitchell et al. demonstrated an immune response to H parainfluenza in patients with COPD, suggesting the organism's pathogenicity [5]. However, Sethi et al. revealed a non-significant inflammatory response to H. parainfluenzae in patients with COPD [6]. Hemophilus parainfluenza was found to cause pneumonia in an otherwise healthy individual with a demonstrated antibody response [7]. In our presented case, the patient exhibited intermittent well-controlled asthma, hypertension, and obesity as concurrent comorbidities. Contrasting with previously documented instances of Hemophilus influenza-associated empyema accompanied by pneumonia, one patient suffered from chronic obstructive pulmonary disease (COPD) and alcoholism [3], while another had rheumatoid arthritis and was undergoing active treatment [4]. Notably, our patient's QuantiFERON test yielded a positive result; however, no pulmonary infiltrates were discerned on chest imaging. Moreover, a negative mycobacterial culture of the pleural fluid pointed toward latent tuberculosis or prior BCG vaccination as plausible explanations.

A study conducted in Spain aimed to ascertain the antibiotic susceptibilities of Hemophilus species, uncovering that Hemophilus parainfluenza displayed greater resistance to non-beta-lactam antimicrobials [8]. Soriano et al. documented the effectiveness of Levofloxacin, cefotaxime, cefpodoxime, cefuroxime, amoxicillin/clavulanate, and clarithromycin against Hemophilus parainfluenza [9]. In our patient's case, ceftriaxone was initially administered, followed by subsequent treatment with amoxicillin/clavulanic acid, which elicited an appropriate and satisfactory response.

## Conclusions

Our patient exhibited an atypical, asymptomatic presentation that notably lacked underlying pneumonia, ultimately culminating in the isolation of Hemophilus parainfluenza from the pleural fluid. Subsequent antibiotic treatment yielded a positive response. This case underscores the significance of recognizing Hemophilus parainfluenza as a potential causative agent in pleural effusion cases.
